# Hedgehog Signaling in Endochondral Ossification

**DOI:** 10.3390/jdb4020020

**Published:** 2016-06-03

**Authors:** Shinsuke Ohba

**Affiliations:** Department of Bioengineering, Graduate School of Engineering, The University of Tokyo, 7-3-1 Hongo, Bunkyo-ku, Tokyo 113-0033, Japan; ohba@bioeng.t.u-tokyo.ac.jp; Tel.: +81-3-5841-1427; Fax: +81-3-5841-1428

**Keywords:** Indian hedgehog (Ihh), endochondral ossification, chondrocyte, osteoblast

## Abstract

Hedgehog (Hh) signaling plays crucial roles in the patterning and morphogenesis of various organs within the bodies of vertebrates and insects. Endochondral ossification is one of the notable developmental events in which Hh signaling acts as a master regulator. Among three Hh proteins in mammals, Indian hedgehog (Ihh) is known to work as a major Hh input that induces biological impact of Hh signaling on the endochondral ossification. Ihh is expressed in prehypertrophic and hypertrophic chondrocytes of developing endochondral bones. Genetic studies so far have demonstrated that the Ihh-mediated activation of Hh signaling synchronizes chondrogenesis and osteogenesis during endochondral ossification by regulating the following processes: (1) chondrocyte differentiation; (2) chondrocyte proliferation; and (3) specification of bone-forming osteoblasts. Ihh not only forms a negative feedback loop with parathyroid hormone-related protein (PTHrP) to maintain the growth plate length, but also directly promotes chondrocyte propagation. Ihh input is required for the specification of progenitors into osteoblast precursors. The combinatorial approaches of genome-wide analyses and mouse genetics will facilitate understanding of the regulatory mechanisms underlying the roles of Hh signaling in endochondral ossification, providing genome-level evidence of the potential of Hh signaling for the treatment of skeletal disorders.

## 1. Introduction

Hedgehog (Hh) signaling plays crucial roles in the patterning and morphogenesis of various organs within the bodies of vertebrates and insects [1]. In the conventional model of Hh signal transduction, the seven-pass transmembrane protein Smoothened (Smo) has an intrinsic intracellular signaling activity, which is repressed by the 12-pass transmembrane Hh receptor Patched (Ptch). The binding of Hh ligands to Ptch initiates Hh-Ptch signal transduction by relieving the repressive effect of Ptch on Smo. In vertebrates, three zinc finger transcription factors Gli1, Gli2, and Gli3 mediate Hh-responsive transcription [1]. Gli1 acts as a strong transcriptional activator. Gli2 and Gli3 are processed into transcriptional repressor forms upon Hh input. Gli2 has been suggested to function primarily as a transcriptional activator, and Gli3 as a transcriptional repressor, although a few studies have shown the opposite (for review see [1]).

Substantial progress has been made in understanding how Hh signaling engages skeletal development and interacts with other signaling factors, mainly based on mouse genetic studies manipulating the Hh signaling molecules mentioned above. This review aims to summarize the roles of Hh and its mode of action in skeletal development, with a particular focus on endochondral ossification.

## 2. Indian Hedgehog (Ihh) Synchronizes Chondrogenesis and Osteogenesis during Endochondral Ossification

Mammalian skeletons are formed by two distinct processes: intramembranous ossification and endochondral ossification. In intramembranous ossification, mesenchymal cells directly differentiate into the bone-forming cells called osteoblasts. This process forms the frontal bone, parietal bone, maxilla, mandibular bone, part of the clavicle, and so on. In contrast, axial skeletons and the long bones in the limbs develop through endochondral ossification, a process in which cartilage molds are initially formed and replaced by bones. During the endochondral ossification process, cartilage formation (chondrogenesis) and bone formation (osteogenesis) are orchestrated in a spatially and temporally controlled manner.

Endochondral ossification starts with the condensation of immature mesenchymal cells at a region where future bone develops. Cells in the center of the condensation give rise to chondrocytes expressing cartilage matrixes, including type II collagen, whereas cells at the periphery of the condensation form the perichondrium. Cartilage tissues grow through the proliferation and maturation of chondrocytes. Chondrocyte maturation is represented by its post-mitotic hypertrophy and the deposition of calcified matrices. Hypertrophic chondrocytes undergo apoptosis, followed by the absorption of residual calcified matrix by chondroclasts that come into the skeleton upon vascular invasion.

Cells at a certain region in the perichondrium (see later) are specified into an osteoblast lineage to form the bone collar, a predecessor of cortical bones. Osteoblasts are generated through several precursors. Immature mesenchymal cells initially differentiate into osteoblast precursors expressing runt-related transcription factor 2 (Runx2); the Runx2-positive precursors then express Sp7 (osterix) as well as Runx2, further differentiating into mature osteoblasts. Runx2 and Sp7 are key determinants for the osteoblast lineage. Mature osteoblasts contribute to bone formation by secreting bone matrixes. Bone gla protein (Bglap, osteocalcin) is a bona-fide marker for mature osteoblasts.

**Figure 1:**
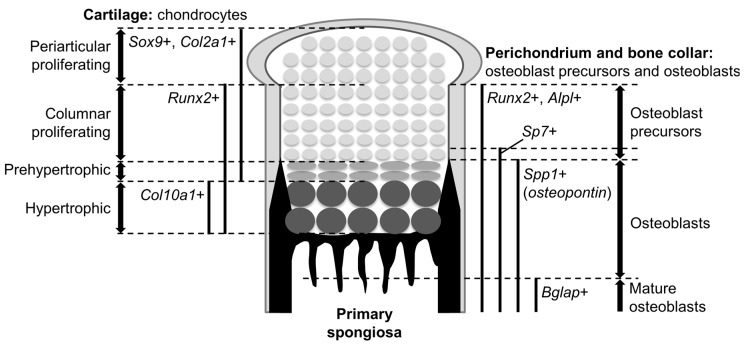
Structure of the growth plate in mouse embryos. An epiphysis of a fetal mouse long bone is depicted. The expression domains of marker genes and the locations of different populations are indicated (left, cartilage; right, perichondrium-bone collar).

In the fetal cartilage of endochondral skeletons, referred to as the growth plate hereafter, chondrocytes are sequentially layered and aligned according to their differentiation phases: the proliferating phase, prehypertrophic phase, and hypertrophic phase (Figure 1). Bone collar formation in the perichondrium synchronizes with chondrocyte hypertrophy; it occurs at the perichondrial region adjacent to hypertrophic chondrocytes (Figure 1). Thus, the process of chondrocyte maturation and osteoblast differentiation can be observed in their chronological order from the epiphysis toward the diaphysis (Figure 1).

**Figure 2:**
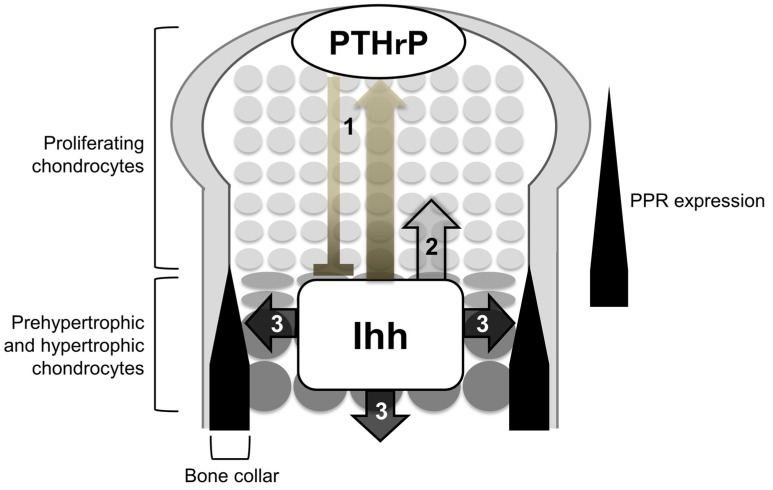
Roles of Ihh in endochondral ossification. Ihh is expressed in prehypertrophic and hypertrophic chondrocytes, regulating the following three processes: (1) chondrocyte differentiation; (2) chondrocyte proliferation; and (3) specification of bone-forming osteoblasts. (1) Ihh secreted from prehypertrophic and hypertrophic chondrocytes induces PTHrP in the periarticular regions including periarticular chondrocytes and the perichondrium. PTHrP then suppresses the transition of columnar proliferating chondrocytes into hypertrophic chondrocytes via PPR (bold line). PPR is expressed strongly in prehypertrophic chondrocytes and weakly in proliferating chondrocytes; (2) Ihh directly stimulates the proliferation of chondrocytes; (3) Ihh is required for the osteoblast differentiation of cells in a population of the perichondrium and primary spongiosa adjacent to Ihh-producing prehypertrophic and hypertrophic chondrocytes.

Among the three Hh proteins in mammals, Ihh is known to work as a major Hh input that induces biological impact of Hh signaling on the endochondral ossification. In developing endochondral bones, prehypertrophic and hypertrophic chondrocytes in the growth plate express the Ihh gene. Target genes of Hh signaling, Ptch1 and Gli1, are expressed in proliferating chondrocytes, perichondrial cells, and primary spongiosa. Cells closer to the Ihh-producing cells express the target genes at higher levels. Genetic studies so far have demonstrated that the Ihh-mediated activation of Hh signaling synchronizes chondrogenesis and osteogenesis during endochondral ossification by regulating the following processes (Figure 2): (1) chondrocyte differentiation; (2) chondrocyte proliferation; and (3) specification of bone-forming osteoblasts; the first action is suggested to be primarily exerted in an indirect manner via parathyroid hormone-related protein (PTHrP) (see below), whereas the second and third ones are exerted in a direct manner.

## 3. Ihh Controls both Differentiation and Proliferation of the Growth Plate Chondrocytes

In 1996, two papers by Lanske *et al.* and Vortkamp *et al.* initially proposed possible roles of Hh signaling in skeletal development in relation to the parathyroid hormone-related protein (PTHrP) [2,3]. PTHrP is expressed in periarticular proliferating chondrocytes and the perichondrium, whereas its receptor, parathyroid hormone 1 receptor (PTH1R; also described as the PTH/PTHrP receptor, or PPR), is expressed strongly in prehypertrophic chondrocytes and weakly in proliferating chondrocytes [4,5]. A series of loss-of-function studies in mice with the deletion of PTHrP, PPR, or both PTH and PTHrP [2,6,7,8] and gain-of-function studies in mice with the chondrocyte-specific overexpression of PTHrP or the constitutively active PPR [9,10,11] indicated that PTHrP kept chondrocytes proliferating by suppressing their hypertrophy via PPR.

In the paper by Vortkamp *et al.*, Ihh overexpression in chick limbs decreased hypertrophic chondrocytes while increasing PTHrP expression in the periarticular perichondrium [3]. Although premature hypertrophy of *PTHrP*^−/−^ limbs was rescued by the addition of PTHrP, Shh had no effect on the *PTHrP*^−/−^ limbs. By utilizing mouse limb explant cultures, Lanske *et al.* also showed that although PTHrP treatment and Shh treatment both induced the elongation of the growth plate with the suppression of chondrocyte hypertrophy in wild-type limbs, neither of them had effects on *PPR*^−/−^ limbs [2]. Together with the findings on PTHrP function mentioned in the last paragraph, the data presented by Vortkamp *et al.* and Lanske *et al.* suggested that the transition from proliferating chondrocytes to hypertrophic ones was regulated by both PTHrP-PPR signaling and Ihh in a common feedback loop, where the PTHrP-PPR signaling mediated the effect of Ihh on the transition.

Skeletal phenotypes of *Ihh^−/−^* mice were then reported in 1999 [12]. *Ihh^−/−^* mice showed shortening of the proliferating chondrocyte layer and acceleration of chondrocyte hypertrophy, as seen in the loss of function of PTHrP signaling in mice. More importantly, these phenotypes accompanied a loss of PTHrP expression in the periarticular regions. In contrast, when Hh signaling was activated specifically in chondrocytes by the loss of *Ptch1* in mice, chondrocyte hypertrophy was delayed and PTHrP expression was upregulated in the periarticular regions [13]. These phenotypes partly supported the hypothesis on the interaction between Ihh and PTHrP.

The interaction was genetically determined by Karp *et al.* through the analysis of compound mutant mouse lines [14]. *Ihh*^−/−^; *PTHrP*^−/−^ mutant limbs showed abnormalities similar to *Ihh*^−/−^ mutant limbs; both mutants exhibited premature chondrocyte hypertrophy and the absence of the growth plate, trabecular bone, and bone collar. Karp and colleagues next activated PTHrP signaling in *Ihh*^−/−^ chondrocytes in order to clarify which functions of Ihh were mediated by PTHrP. The compound mutants inhibited premature chondrocyte hypertrophy, but did not increase the number of mitotically active chondrocytes compared to *Ihh*^−/−^ mutants. These results suggest that: (1) Ihh is required for both the differentiation and the proliferation of growth plate chondrocytes; (2) PTHrP mediates part of the function of Ihh to regulate the maintenance of a pool of proliferating chondrocytes; and (3) Ihh can promote the proliferation of these cells in a PTHrP-independent manner.

Growth plate phenotypes of chimeric mice carrying *PPR*^−/−^ cells and WT cells further provided a clue about the interaction between Ihh and PTHrP [15] (Table 1). Embryonic stem (ES) cell lines homozygous for the *PPR*-null mutation (*PPR*^−/−^ cells) were injected into WT blastocysts to produce the chimera [15]. *PPR*^−/−^ chondrocytes ectopically became hypertrophic in columnar proliferating regions and expressed Ihh. The ectopic hypertrophy of *PPR*^−/−^ cells was accompanied by the upregulation of PTHrP expression in periarticular WT cells and an elongation of the growth plate. Chimeric mice carrying *PPR*^−/−^; *Ihh^−/−^* cells and WT cells revealed the role of Ihh in the interaction [16]. Although *PPR*^−/−^; *Ihh^−/−^* chondrocytes still exhibited ectopic hypertrophy in the manner of the *PPR*^−/−^ chondrocytes, the upregulation of PTHrP expression and the elongation of the growth plate were no longer observed in the chimeras.

Thus, the genetic studies introduced so far support the idea that Ihh constitutes a negative feedback loop with PTHrP to maintain the growth plate length (Figure 2): Ihh secreted from prehypertrophic chondrocytes induces the expression of PTHrP in the periarticular regions including periarticular chondrocytes and the perichondrium, possibly in a concentration-dependent manner as morphogens typically do. PTHrP suppresses chondrocyte hypertrophy by acting on prehypertrophic chondrocytes that strongly express PPR. The suppression of hypertrophy keeps chondrocytes proliferating, and the distance between PTHrP-producing cells and Ihh-producing cells increases. To correct for this change in the distance, a change in the expression of PTHrP is induced. Thus, the feedback loop maintains a pool of proliferating chondrocytes, and thereby maintains a certain length of the growth plate in order to maximize skeletal growth. In this context, direct Ihh input is likely required for PTHrP expression in periarticular proliferating chondrocytes, since the local removal of *Smo* caused little or no expression of PTHrP within the periarticular domain that showed no activation of Hh signaling [17]. There are also evidences for the induction of the PTHrP expression by Ihh in the perichondrium. As noted earlier, the study by Vortkamp *et al.* demonstrated that Ihh overexpression increased PTHrP expression in the periarticular perichondrium [3], suggesting that Ihh acts on the perichondrium to express PTHrP, *i.e.*, the initiation of the feedback loop. In addition, removal of the perichondrium from chicken embryonic tibiotarsi resulted in the expansion of the hypertrophic chondrocyte layer; the addition of parathyroid hormone, which binds to PPR as PTHrP does, cancelled the expansion [18]. These finding suggest PPR-dependent roles of the perichondrium in the regulation of cartilage differentiation. The study by Long *et al.*, together with the one by Vortkamp *et al.*, may further support the importance of the perichondrium as a source of PTHrP in the negative feedback loop.

In addition to its indirect action on chondrocyte differentiation via PTHrP, Ihh has been shown to have direct actions on growth plate chondrocytes. Decreased proliferation of chondrocytes was observed in *Ihh^−/−^* mice and chondrocyte-specific *Smo^−/−^* mice [12,19], indicating that Ihh input directly promotes chondrocyte propagation in the growth plate as suggested by Karp *et al.* (see earlier and Figure 2) [14]. In addition, two studies have suggested that Ihh can also directly regulate the differentiation of growth plate chondrocytes at multiple steps (periarticular proliferating to columnar proliferating; columnar proliferating to hypertrophic) in a PTHrP-independent manner. Kobayashi *et al.* proposed that Ihh promoted the differentiation of periarticular proliferating chondrocytes into columnar proliferating chondrocytes independently of PTHrP [20,21], while Mak *et al.* showed that chondrocyte-specific removal of Smo delayed chondrocyte hypertrophy without PTHrP in mice [22].

Which Gli factors mediate the biological effects of Ihh on the growth plate chondrocytes? Gli3 is likely to primarily act upon Ihh input in this context. Abnormalities in the proliferation and maturation of chondrocytes in *Ihh^−/−^* mice were rescued by removal of *Gli3* on an *Ihh^−/−^* background, accompanying the recovery of PTHrP expression in periarticular regions [23,24]. In contrast, no obvious defects in the growth plate were observed in either *Gli1^−/−^*, *Gli2^−/−^* or *Gli1^−/−^*; *Gli2^−/−^* mouse embryos [8,25,26,27]. Thus, the suppression of Gli3 repressor activity by Ihh plays more dominant roles in the maintenance of the growth plate than the activation of Gli activators by Ihh.

Several studies shed light on postnatal roles of Hh signaling in cartilage and the growth plate. Postnatal deletion of *Ihh* from *Col2a1*-expressing cells caused ectopic chondrocyte hypertrophy, decreased proliferation of chondrocytes, and the disorganized growth plate in mice [28]. When *Ihh* was deleted from *Prrx1*-expressing skeletal progenitors, the growth plate and secondary ossification center were absent at postnatal stages in mice [29]. These data indicate the postnatal requirement of Ihh during endochondral bone development. Maeda *et al.* further showed that Ihh functioned in both PTHrP-dependent and -independent manners in this context [30]. The ectopic chondrocyte hypertrophy led by the deletion of *Ihh* from *Col2a1*-expressing cells was temporally rescued by the forced expression of constitutively active *PPR* in the *Ihh-*deleted population using a *Col2a1* promoter fragment, whereas decreased proliferation of chondrocytes was not corrected in the compound mutant. These data suggest that: (1) Ihh has positive effects on chondrocyte proliferation at the postnatal stage, but the effects are unlikely mediated by PPR; and (2) Ihh and PTHrP may functionally interact at postnatal stages, as they do at embryonic stages. However, given the temporal rescue of growth plate phenotypes in the compound mutant, contributions of the interaction to chondrocyte hypertrophy, if any, are likely to be less at postnatal stages than at embryonic stages; other mechanisms or signaling molecules may also mediate the process. Hirai *et al.* showed that chondrocyte-specific ablation of *PPR* in postnatal mice led to the acceleration of hypertrophy, followed by premature closure of the growth plate, in association with increased chondrocyte apoptosis [31]. They suggest the possibility that the above mechanism may also underlie the growth plate closure caused by the postnatal deletion of *Ihh* from *Col2a1*-expressing cells, because Ihh induces PTHrP.

## 4. Ihh is Required for Osteoblast Differentiation

Ihh is a master regulator of osteoblast specification during endochondral ossification. In endochondral ossification, osteoblasts first appear in a region of the perichondrium adjacent to pre-hypertrophic and hypertrophic chondrocytes. The following mouse genetic studies have indicated that direct Ihh input from these chondrocytes to progenitors is required for osteoblast specification in the perichondrium and the primary spongiosa. Both bone collars and the expression of Runx2 and Bglap were absent in the perichondrium of *Ihh^−/−^* mice [12,23]. Involvement of Ihh and Ihh-producing cells, *i.e.*, pre- and hypertrophic chondrocytes, in bone collar formation in the perichondrium was further supported by phenotypes of *PPR*^−/−^/WT chimeric mice and *PPR*^−/−^; *Ihh^−/−^*/WT chimeric mice [15,16] (Table 1). *PPR*^−/−^; *Ihh^−/−^*/WT chimeric mice were generated by the injection of *PPR*^−/−^; *Ihh^−/−^* ES cells into WT blastocysts [16]. In *PPR*^−/−^/WT chimeras, perichondrial cells adjacent to ectopically hypertrophic *PPR*^−/−^ cells underwent ectopic calcification. The ectopic calcification was cancelled by the removal of *Ihh* in the ectopically hypertrophic *PPR*^−/−^ cells, although ectopic hypertrophy was still observed in the *PPR*^−/−^; *Ihh^−/−^* cells with robust expressions of bone morphogenetic proteins (BMP) 2 and 6. Thus, Ihh produced by pre-hypertrophic and hypertrophic chondrocytes is necessary for the differentiation of perichondrial cells into osteoblasts.

When Hh signaling activity was removed by the *Smo* deletion in perichondrial cells, Runx2 expression and bone collar formation were completely absent in the perichondrium [32]. In *Smo^−/−^*/WT chimeric mice, *Smo^−/−^* perichondrial cells in the region where the bone collar is formed under physiological conditions expressed chondrocyte marker genes including type II collagen (Col2a1) and type X collagen (Col10a1), and did not undergo osteoblast differentiation; *Smo^−/−^* cells in the primary spongiosa did not contribute to bone formation [32]. Chondrocyte-specific overexpression of *Ihh* or the ablation of *Ptch1* from perichondrial cells, which caused supra-physiological activation of Hh signaling in each population, resulted in the accelerated bone collar formation [13,32].

Closer analyses of *Ihh^−/−^* mice by Colnot *et al.* provide clues regarding the mechanism by which Hh signaling regulates the osteoblast differentiation of perichondrial cells at the cellular level [33]. They found that perichondrial cells are thin and disorganized in *Ihh^−/−^* mice; the abnormality became evident within 24 h after the physiological onset of Ihh expression at E12.5. In E14 *Ihh^−/−^* embryos, *Col1a1* expression was diffuse and not restricted to cells adjacent to *Col2a1*-expressing chondrogenic condensation in the perichondrial region. Given that Runx2 appears at E12.5 in a physiological context, the organization of perichondrial cells may be associated with the cell fate specification of progenitors into Runx2-positive osteoblast precursors.

Thus, Ihh produced by pre- and hypertrophic chondrocytes is essential for osteoblast development in the perichondrium and the primary spongiosa during endochondral ossification (Figure 2); more specifically, Hh signaling is required for the specification of progenitors into Runx2-positive osteoblast precursors. Differentiation of the Runx2-positive osteoblast precursors into Sp7-positive ones is unlikely to require Hh signaling, since the ablation of *Smo* from *Sp7*-expressing cells does not affect osteoblast differentiation [34]. Given that perichondrial cells that cannot receive Hh input acquire chondrocytic phenotypes [32], the signaling may act as a molecular switch for the cell fate specification of the osteo-chondroprogenitor population.

We and others have shown that Hh signaling regulates not only skeletal development, but also postnatal bone homeostasis. Disruption of Ihh in postnatal chondrocytes led to premature vascularization, a delay in the formation of the ossification center, and less trabecular bone [28], suggesting that chondrocyte-derived Ihh was involved in the maintenance of growth plates and trabecular bones in the postnatal stage. *Ptch1^+/−^* mice had high bone mass with an acceleration of both bone formation and bone resorption; a similar trend to that in the mouse phenotypes was observed in patients with basal cell carcinoma syndrome (BCCS) caused by an inactivating mutation of one of the *Ptch1* alleles [35]. Although high bone turnover phenotypes were also observed in mice in which *Ptch1* was disrupted in *Bglap*-expressing mature osteoblasts, they showed low bone mass unlike *Ptch1^+/−^* mutants [36]; it is possible that the indirect effects of Hh signaling on osteoclasts are dominant over its direct effects on osteoblasts, when Hh signaling was activated specifically in mature osteoblasts. In addition, heterozygous deletion of *Gli1* led to decreased bone mass with an uncoupling of bone formation and resorption in mice, suggesting that Gli activators participate in postnatal bone homeostasis upon Hh input [37].

## 5. Hierarchy between Hh Signaling and Other Osteogenic Factors in Osteoblast Differentiation

Genetic studies may suggest a further conclusion about the hierarchy between Hh signaling and other osteogenic signaling factors such as Wnt and bone morphogenetic proteins (BMPs) during osteoblast differentiation. The differentiation was largely arrested at the Runx2-positive stage, when canonical Wnt signaling activity was removed in the limb bud [38], the mesenchymal condensation [39,40], or the perichondrium [34,40]; the removal of canonical Wnt signaling activity in *Sp7*-expressing osteoblast precursors resulted in a lack of *Bglap*-expressing mature osteoblasts [34]. These data indicate that canonical Wnt signaling is required for the transition of Runx2-positive osteoblast precursors into Sp7-positive ones and for the transition of Sp7-positive precursors into mature osteoblasts, both of which occur later than the phase requiring Hh signaling. Indeed, canonical Wnt signaling was impaired in *Ihh^−/−^* mice [39], and the activation of Hh signaling in the perichondrium did not rescue the reduction of bone formation in mice defective for canonical Wnt signaling activity [40].

By taking advantage of *ex vivo* organ cultures as well as mouse genetics, we analyzed the hierarchy between Hh and BMP signaling in skeletal development [27]. The activation of BMP signaling accelerated both osteogenesis and chondrogenesis in the perichondrium, but only after Hh-dependent lineage specification into osteoblasts or chondrocytes took place [27]. In the physiological context, osteogenic function of BMP is likely to require Hh-Gli activator-mediated specification of progenitors into osteoblasts in the perichondrium; BMP can act as a chondrogenic factor on the perichondrial cells only when they do not receive Hh input [27].

Thus, Hh signaling acts epistatically on both canonical Wnt signaling and BMP signaling in the osteoblast differentiation cascade, and these pathways sequentially regulate the cascades. Genetic studies so far collectively support the following model for osteoblast differentiation and maturation: Skeletal progenitors are specified into the Runx2-positive osteoblast precursor upon Hh input [12,32]. Canonical Wnt signaling then sequentially induces both the transition of the Runx2-positive osteoblast precursor into the Sp7-positive one [38,39,40] and the generation of Bglap (bone gla protein)-positive, bone-forming osteoblasts from the Sp7-positive precursor [34]. BMP signaling is likely to accelerate a series of these processes after the Hh-mediated specification into an osteoblast lineage [27]. Mutant mice where *Bmp2* and *Bmp4* were deleted in *Prrx1*-expressing skeletal progenitors showed comparable expression of Runx2 to WT mice [41], but lost Sp7 expression at 3 weeks of age, suggesting that BMP signaling may be involved in the transition of the Runx2-positive osteoblast precursor into the Sp7-positive one, which occurs later than the phase requiring Hh signaling. Given that decapentaplegic (dpp), a homolog of BMP has been shown to act downstream of hedgehog in Drosophila (reviewed in [1]), one may think that BMP signaling mediates the action of Ihh in mammalian osteoblast development. BMP2 and BMP6 are expressed in hypertrophic chondrocytes as Ihh is in developing endochondral bones [16]. When the ectopic calcification in the perichondrium was cancelled by the removal of *Ihh* in the ectopically hypertrophic *PPR*^−/−^ cells as mentioned earlier (Table 1), BMP2 and BMP6 were still expressed in the ectopically hypertrophic *PPR*^−/−^ cells [16]. This result provides a genetic support for that BMP signaling is unlikely to act downstream of Hh signaling in mammalian osteoblast development.

## 6. Molecules Acting Downstream of Hh Input in Osteoblast Differentiation

Regarding molecules mediating the biological actions of Hh signaling on osteoblast differentiation, it seems reasonable to imagine that Gli and Runx2 are major mediators of these actions, given that Gli acts downstream of Hh signaling and that Runx2 expression is lost in the perichondrium of mice lacking Hh signaling.

A series of studies indicated that Gli1, Gli2, and Gli3 are involved in Hh-mediated osteoblast differentiation. Shimoyama *et al.* showed that Ihh promoted osteoblast differentiation in a Gli2-dependent manner; and Ihh treatment or Gli2 overexpression induced the expression of Runx2, which physically interacted with Gli2 [42]. In line with this, the skeletal phenotypes of *Ihh*^−/−^ embryos were completely rescued in *Ihh*^−/−^; *Gli3*^−/−^; *C2*-*NGli2* embryos, in which *NGli2* (an N-terminally truncated, constitutively active form of Gli2) was exogenously expressed in *Col2a1*-expressing cells [43]. On the basis of these findings, Joeng and Long proposed that the Gli2 activator and the Gli3 repressor collectively mediated all major aspects of Ihh function in endochondral ossification.

We recently found that bone formation was impaired in *Gli1*^−/−^ mice; *Gli1*^−/−^ perichondrial cells expressed Col2a1 and Col10a1, but not Runx2 or Sp7. *Gli1*^−/−^; *Gli2*^−/−^ mice showed more severe skeletal phenotypes than either *Gli1*^−/−^ or *Gli2*^−/−^ mice, and osteoblast differentiation was impaired in *Gli1*^−/−^; *Gli3*^−/−^ perichondrial cells compared to that in *Gli3*^−/−^ cells *in vitro* [26]. Indeed, Gli1 activated the transcription of early and middle marker genes for osteoblasts by directly binding to 5′ regulatory regions of the genes [26], and it interfered with the Sox9-mediated transactivation of chondrocyte marker genes by suppressing the DNA binding of Sox9 [27]. We also found that the Gli3 repressor suppressed the Runx2-mediated transcription of osteoblastic genes by antagonizing the DNA binding of Runx2 [35]. These data suggest that Gli1 functions collectively with Gli2 and Gli3 in osteoblast formation, particularly in the specification of progenitors into osteoblasts.

Regarding the involvement of Runx2 in the action of Hh, the recovery of Runx2 expression in the *Ihh^−/−^* perichondrium did not cancel the abnormality of osteoblast differentiation in *Ihh^−/−^* mice [44], suggesting that Hh signaling used effectors other than *Runx2* to promote osteoblast differentiation. Gli transcription factors are possible candidates for these effectors.

Recently, Regard *et al.* found that an abnormal activation of Hh signaling underlay heterotopic ossification, which is caused by the inactivating mutation of *Gnas* [45]. GNAS encodes Gαs, a transducer of the G protein-coupled receptor (GPCR); Gαs was suggested to act downstream of Smo and upstream of Gli, and the Gαs-cAMP-PKA axis suppressed Hh signaling activities by regulating Gli activation and processing in this context. Taken together, these facts suggested that Gαs negatively modulated the Hh signaling activity [45].

## 7. Conclusions

As summarized in this review, over the last two decades mouse genetic approaches have unraveled the functions of Hh signaling and their underlying molecular mechanisms in endochondral ossification. Ihh regulates chondrocyte differentiation by constituting a negative feedback loop with PTHrP, which is a central system in the proper growth of endochondral skeletons; Ihh plays a prerequisite role in osteoblast formation. However, the gene regulatory networks underlying these actions are still poorly understood. In particular, what genes are regulated by Gli factors and translate Hh input into the biological actions on skeleton? What transcription factors engage the Gli-mediated network during skeletal development? A combinatorial approach using genome-wide analyses and mouse genetics will facilitate understanding of these regulatory mechanisms, providing genome-level evidence of the potential of Hh signaling for the treatment of skeletal disorders.

## Figures and Tables

**Table 1:** Comparison of phenotypes between *PPR*^−/−^/WT chimeric and *PPR*^−/−^; *Ihh^−/−^*/WT chimeric.mice.

	*PPR*^−/−^/WT Chimeras	*PPR*^−/−^; *Ihh^−/−^*/WT Chimeras
Ectopic hypertrophy of mutant cells	+	+
Upregulation of PTHrP in periarticular WT cells	+	−
Elongation of the growth plate	+	−
Ectopic bone collar formation in the perichondrum	+	−
